# Measuring the Non-linear Directed Information Flow in Schizophrenia by Multivariate Transfer Entropy

**DOI:** 10.3389/fncom.2019.00085

**Published:** 2020-01-10

**Authors:** Dennis Joe Harmah, Cunbo Li, Fali Li, Yuanyuan Liao, Jiuju Wang, Walid M. A. Ayedh, Joyce Chelangat Bore, Dezhong Yao, Wentian Dong, Peng Xu

**Affiliations:** ^1^The Clinical Hospital of Chengdu Brain Science Institute, MOE Key Lab for Neuroinformation, University of Electronic Science and Technology of China, Chengdu, China; ^2^School of Life Science and Technology, Center for Information in Medicine, University of Electronic Science and Technology of China, Chengdu, China; ^3^Institute of Mental Health, Peking University Sixth Hospital, National Clinical Research Center for Mental Disorders & Key Laboratory of Mental Health, Ministry of Health, Peking University, Beijing, China

**Keywords:** network deterioration, schizophrenia, non-linear causal interaction, multivariate transfer entropy, granger causality, bivariate transfer entropy

## Abstract

People living with schizophrenia (SCZ) experience severe brain network deterioration. The brain is constantly fizzling with non-linear causal activities measured by electroencephalogram (EEG) and despite the variety of effective connectivity methods, only few approaches can quantify the direct non-linear causal interactions. To circumvent this problem, we are motivated to quantitatively measure the effective connectivity by multivariate transfer entropy (MTE) which has been demonstrated to be able to capture both linear and non-linear causal relationships effectively. In this work, we propose to construct the EEG effective network by MTE and further compare its performance with the Granger causal analysis (GCA) and Bivariate transfer entropy (BVTE). The simulation results quantitatively show that MTE outperformed GCA and BVTE under varied signal-to-noise conditions, edges recovered, sensitivity, and specificity. Moreover, its applications to the P300 task EEG of healthy controls (HC) and SCZ patients further clearly show the deteriorated network interactions of SCZ, compared to that of the HC. The MTE provides a novel tool to potentially deepen our knowledge of the brain network deterioration of the SCZ.

## Introduction

The brain usually fizzles with the non-linear causal activity of electroencephalogram (EEG) at a microscopic level (Gourévitch et al., 2006; Sabesan et al., 2010; Mehta and Kliewer, 2018). The complex nature of the brain makes its non-linear causal dynamics unknown, and how the brain matches its rhythm as well as its metabolic processes and a causal relationship is still under investigation. The brain might be attacked with many psychosomatic diseases such as schizophrenia (SCZ), leading to deteriorated brain network, which eventually affects its cognitive functions (Shovon et al., 2017; Li et al., 2018). Researchers have explored the EEG non-linearity in multiple psychiatric disorders, for example, in epileptic patients probably due to low dimensional chaos during a seizure (Lee et al., 2001; Henderson et al., 2011; Liu et al., 2017). Thus, the behavioral and psychological attitudes of people with psychiatric disorders call for the need to effectively investigate the transient information exchange in the brain (Zhang et al., 2011; Mehta and Kliewer, 2016). Multiple techniques or measures for linear and non-linear brain connectivity such as structural, functional, and effective connectivity are in use for this purpose (Selskii et al., 2017; Hristopulos et al., 2019). Exploring the linear and non-linear interactions, more importantly when the system structure is unknown, holds promise for deepening the knowledge of the causal mechanism in the brain for the SCZ (Pereda et al., 2005; Zhao et al., 2013).

SCZ is the most prevalent functional psychotic disorder, and people living with the disorder can present with a variety of symptoms and manifestations that can be seen in their behaviors. The disease is a chronic psychotic disorder that disrupts the patient's thoughts and affect their total well-being (Patel et al., 2014; Ure et al., 2018). Previous studies have demonstrated a coherent or uniform reduction in the brain regions of the SCZ patients, including the insula, superior temporal gyrus, amygdala, parahippocampus, inferior and medial frontal gyri, hippocampus, and anterior cingulate cortex (ACC) (Ehrlich et al., 2014; Alonso-Solís et al., 2015; Domínguez-Iturza et al., 2018). In neurophysiological research, it is more interesting to explore the specific performance of the SCZ under certain task like oddball paradigm involving the P300 (Alvarado-González et al., 2016), as the P300 serves as the reliable biomarker to identify the SCZ from healthy control (HC) (Somani and Shukla, 2012). For example, during working memory, the P300 amplitude decreases with increasing the load for HC but remains low in all conditions for the SCZ (Gaspar et al., 2011). Besides the P300 amplitude, the occurrence of the SCZ is also accompanied by the abnormal task brain network (Krusienski et al., 2006; Pérez-Vidal et al., 2018). For example, we have previously found a crucial role of the ACC in regulating the P300 (Li et al., 2018), especially a compensatory pathway from the dorsolateral prefrontal cortex to intraparietal sulcus for the SCZ.

Effective connectivity in the brain brings in the element of causal interactions or causation. Consequently, a signal activation in one area of the brain directly causes a change or signal, activation or depression, in another area (Mastrovito et al., 2018; Zhu et al., 2018). Effective connectivity in a domain of data-driven approaches such as Granger causality analysis (GCA) which performs poorly in non-linear context rely on its past to formulate linear causal interactions in the EEG signal (Venkatesh and Grover, 2016; Li et al., 2017). The GCA is initially formulated for linear models and later extended to non-linear systems by applying to local linear models. Despite its success in detecting the direction of interactions in the brain, it either makes assumptions about the structure of the interacting systems or the nature of their interactions and as such, it may suffer from the shortcomings of modeling systems/signals of unknown structure (Lainscsek et al., 2013; Sohrabpour et al., 2016; Bonmati, 2018). Even though much has been achieved with the GCA, a different data-driven approach which involves information theoretic measures like Transfer entropy (TE) may play a critical role in elucidating the effective connectivity of non-linear complex systems that the GCA may fail to unearth (Schreiber, 2006; Madulara et al., 2012; Dejman et al., 2017). Mathematically, the TE uses its entropy to quantitatively infer the coupling strength between two variables (Liu and Aviyente, 2012; Shovon et al., 2017) and has the potential for capturing both the linear and non-linear causal interactions effectively. Thus, TE works in bivariate fashion where information transfer is quantified between all source-target pairs but bivariate analysis has spurious, redundant and synergistic interaction problems (James et al., 2016; Wollstadt et al., 2019).

To quantify the effective connectivity and exploring the corresponding network aberration in the SCZ, the reliable estimation of the brain network seems to be of great urgency. In this work, we used the TE in a multivariate fashion (Lainscsek et al., 2013; Alonso-Solís et al., 2015; Bonmati, 2018), i.e., multiple TE (MTE) (Montalto et al., 2014; Novelli et al., 2019; Wollstadt et al., 2019). The MTE has great ability to handle problems that the GCA and the BVTE cannot, such as spurious or redundant interactions, where multiple sources provide the same information about the target, the MTE also cannot miss synergistic interactions between multiple relevant sources and the target, where these multiple sources jointly transfer more information into the target than what could be detected from examining source contributions individually. The MTE is designed to remove redundancies and capture synergistic interactions and account for all relevant sources of a target, unearth both the linear and non-linear dynamics in the brain; thus making it a powerful tool over GCA and BVTE (Stokes et al., 2018; Wollstadt et al., 2019). Herein, we first proposed to infer the linear and non-linear simulations of the GCA, BVTE, and MTE under various conditions, including varied signal-to-noise(SNR) conditions, edges recovered, sensitivity, and specificity, to explore their performances; thereafter, we also applied both methods to P300 task EEG of the SCZ and HC to investigate the brain network deterioration for the SCZ.

## Transfer Entropy

If a signal *X* directly interacts with signal *Y*, then the past information of *X* should possess ample information that can help predict *Y* beyond the information possessed in the history of *Y* only. That is, there is a Granger-causal interaction from *X* to *Y* (Sørensen and Causality, 2005). The GCA paves a way for the examination of the directed interaction between variables. In essence, GCA is designed to measure the linear coupling among time series, which determines that the GCA can only capture the linear causality well, and may not work for the non-linear cases (Bose et al., 2017). In addition, the neural coupling in the brain is far from the linearity, and the conventional GCA may not capture this hidden coupling in the brain.

To capture the non-linear interactions in the brain, we alternatively used the TE to measure the directed information exchange.

Let *X* = {*x*_1_, *x*_2_…, *x*_*T*_} and *Y* = {*y*_1_, *y*_2_, …, *y*_*T*_} denote the time series of two brain areas with T observations, we define an entropy rate which is the amount of additional information required to represent the value of the next observation of *X* as:

(1) $$\begin{array}{r} h_{1}=-\underset{x_{n}+1,x_{n},y_{n}}{\sum }p\left(x_{n}+1,x_{n},y_{n}\right){\text{log}}_{2}p\left(x_{n}+1|x_{n},y_{n}\right) \end{array}$$

Also, we define another entropy rate assuming that *x*_*n*_ + 1 as:

(2) $$\begin{array}{r} h_{2}=-\underset{x_{n}+1,x_{n},y_{n}}{\sum }p\left(x_{n}+1,x_{n},y_{n}\right){\text{log}}_{2}p\left(x_{n}+1|x_{n}\right) \end{array}$$

Therefore, the TE from *Y* to *X* is given by *h*_2_ − *h*_1_, and this corresponds to information transfer from *Y* to *X*:

(3) $$\begin{array}{l} TE_{Y\to X}=h_{2}-h_{1,}\\ \;=\underset{x_{n}+1,x_{n},y_{n}}{\sum }p\left(x_{n}+1,x_{n},y_{n}\right){\text{log}}_{2}\left(\frac{p\left(x_{n}+1|x_{n},y_{n}\right)}{p\left(x_{n}+1|x_{n}\right)}\right) \end{array}$$

Similarly, we can define the transfer entropy from *X* to *Y* as:

(4) $$\begin{array}{r} TE_{X\to Y}=\underset{y_{n}+1,x_{n},y_{n}}{\sum }p\left(y_{n}+1,x_{n},y_{n}\right){\text{log}}_{2}\left(\frac{p\left(y_{n}+1|x_{n},y_{n}\right)}{p\left(y_{n}+1|y_{n}\right)}\right) \end{array}$$

Then, we compute the TE by writing (3) and (4) using conditional probabilities as:

(5) $$\begin{array}{c} TE_{Y\to X}=\underset{x_{n}+1,x_{n},y_{n}}{\sum }p\left(x_{n}+1,x_{n},y_{n}\right){\text{log}}_{2}\\ \left(\frac{p\left(x_{n}+1,x_{n},y_{n}\right)p\left(x_{n}\right)}{p\left(x_{n},y_{n}\right)p\left(x_{n}+1,x_{n}\right)}\right) \end{array}$$

(6) $$\begin{array}{c} TE_{X\to Y}=\underset{y_{n}+1,x_{n},y_{n}}{\sum }p\left(y_{n}+1,x_{n},y_{n}\right){\text{log}}_{2}\\ \begin{array}{c} \left(\frac{p\left(y_{n}+1,x_{n},y_{n}\right)P\left(y_{n}\right)}{p\left(x_{n},y_{n}\right)p\left(y_{n}+1,y_{n}\right)}\right) \end{array} \end{array}$$

Where *x*_*n*_, and *y*_*n*_, are the stochastic variables obtained by sampling the processes at the present time *n* (Gilmour et al., 2012; Wollstadt et al., 2014; Shao et al., 2015).

TE estimator can detect both linear and non-linear causality. However, because of the bivariate nature of TE, its outcome may infer spurious or redundant causality and may also miss synergistic interactions between multiple relevant sources and the target (Wollstadt et al., 2019). Hence, we need to have a tool or method that can accommodate these challenges. MTE has proven to be a better option to measure both the linear and inherent non-linear brain signals and their causal relationships effectively. Importantly, the MTE is an extension of the TE, which is a direct measure of information transfer between a source and a target process in a dynamic or composite system. Unlike TE, however, MTE does not give spurious, redundant information and also may not miss synergistic interactions (Montalto et al., 2014; James et al., 2016; Wollstadt et al., 2019).

Let at a given instance the dynamic system be composed of a source system *X*, a destination system *Y* and remaining systems $Z={\left\{Z^{k}\right\}}_{k=1,\ldots .M-2}$. Here, we are interested in evaluating the information flow from a source system *X* to a destination system *Y*. Then, MTE models the information flow from the source system to the destination system in the presence of the remaining systems, as shown in Equation (7).

(7) $$\begin{array}{l} TE_{X\to Y|Z}\\ =\sum p\left(y_{1:n,}x_{1:n-1,}z_{1:n-1}\right)\text{log}\frac{p\left(y_{n}|x_{1:n-1,}y_{1:n-1,}z_{1:n-1}\right)}{p\left(y_{n}|y_{1:n-1,}z_{1:n-1}\right)} \end{array}$$

Where *x, y*, and *z* are the state visited by the systems *X, Y*, and *Z* over time. Let *x*_*n*_, *y*_*n*_, and *z*_*n*_ be the stochastic variables obtained by sampling the processes at the present time *n*. Furthermore, we denote *x*_*tn*_ as the vector variable describing all the states visited by *X* from time *t* up to *n* (assuming *n* as the present time and setting the origin of time at *t* = 1, *x*_1:*n*−1_ represents the whole past history of the process *x*).

In our case, the dynamic system is composed of the brain regions, Frontal (F), Parietal (P), Temporal (T), and Occipital (O) lobes. In other words, the source system *X* and the destination system *Y* are the brain regions involved in a given information flow, e.g., it could be F and P or T and O. The information flow between any two brain regions is also affected by the states of remaining brain regions, which are not part of the information flow (Wang et al., 2011; Adhikari and Agrawal, 2013; Anil et al., 2015). Hence, MTE is a good estimator to measure the linear and non-linear directed information flow in the brain.

**Figure 1:**
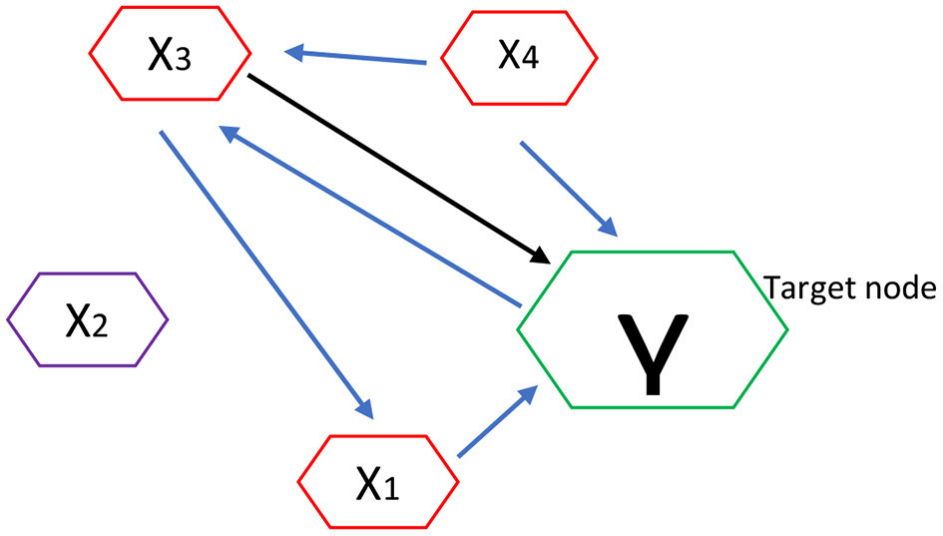
Estimation of MTE into a target node Y. Blue arrows show the estimation of MTE into a target node.

For an illustration, let's demonstrate MTE brain network algorithm analysis as shown in Figure 1. Here the nodes or channels represent (stochastic) processes and the arrows represent causal connections or interactions between processes. It has target of interest and relevant sources.

Thus if *Y* is the current target of interest, then nodes highlighted in red represent the set of relevant sources *Z* = {*X*_1_,*X*_3_,*X*_4_}, i.e., the sources that contribute to the target's current value *Y*_*n*_. In order to estimate the MTE into the target *Y*, it requires inferring the set Z containing the relevant sources (or parents) of *Y*. Once *Z* is inferred, we compute the MTE from a single process into the target as a conditional transfer entropy, which accounts for the potential effects of the remaining relevant sources. Formally, the MTE from a single source (e.g., *X*_3_) into *Y* is defined as the TE from *X*_3_ to *Y*, conditioned on *Z* and excluding *X*_3_: TE(*X*_3_ → *Y*|*Z*\*X*_3_) as shown in Figure 1 (Srivastava, 2002; Flecker et al., 2011; Wollstadt et al., 2019).

## Validation Analysis

### Simulation Study

#### Simulated Network

**Figure 2:**
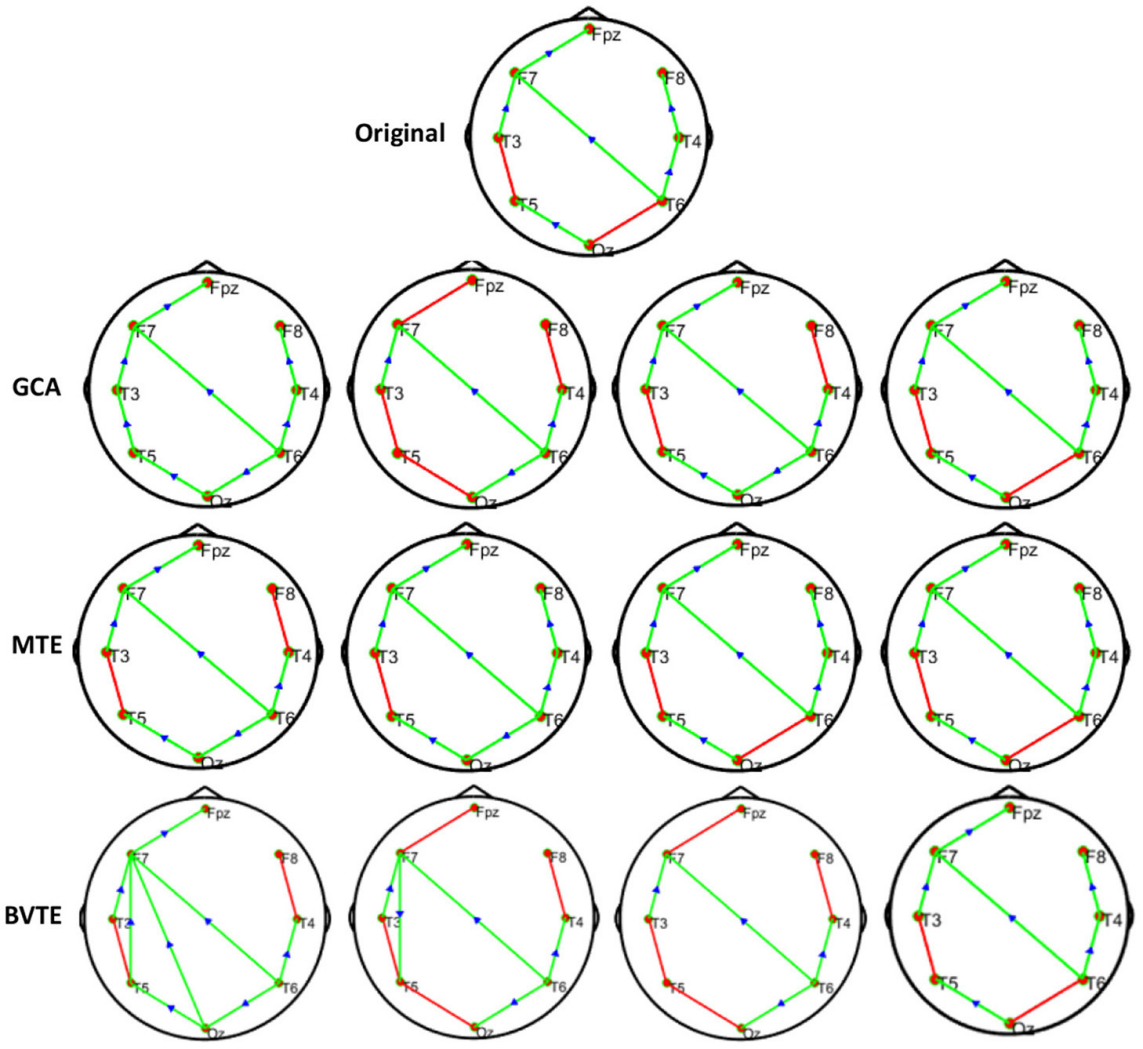
Original or predefined 8 nodes simulated network and estimated linear networks by GCA, MTE, and BVTE with *Y* = *A* × *B*.

We generated and simulated a random time series with 7 and 8 nodes/process and 500 observations (Figures 2, 5). A network structure with unidirectional and bidirectional couplings and nodes with input and output degrees or domain were considered. Two network structures were simulated, i.e., linear and non-linear. Out of the linear equation, we modeled the non-linear networks by adding five different types of non-linear functions to the linear equation (Khadem and Hossein-Zadeh, 2014; Dong et al., 2015; Li et al., 2017). When estimating the MTE and the BVTE, we used the toolbox IDTx (Wollstadt et al., 2019) and GCCA-toolbox for GCA, to estimate the parameters of the MVAR models and the Akaike Information Criterion (AIC) for model order selection (Sohrabpour et al., 2016). We applied the conventional multivariate Granger Analysis for our computation and analysis for GCA. The performance of the GCA, BVTE, and MTE are statistically tested under multiple strategies including the effective connectivity, edges recovered, sensitivity, and specificity on the 8 nodes time series.

To see which method performs better by suppressing the turbulent noise condition, we added Gaussian noise (Ozaki, 2012) with a varying SNR in a range of −10, −5, 5, and 10 dB to the generated time series. With different realizations of the driving noises, each of the network simulations was repeated 200 times for each linear and non-linear equations.

To know the percentage of available causal connections that are correctly detected as existent and the percentage of unavailable causal connections that are really detected as non-existent, the sensitivity and specificity analysis were calculated, respectively. Confusion matrix function is used for the sensitivity and specificity calculations. It is made up of a target matrix and the actual matrix. The confusion matrix compares the relationship between the target matrix and the actual matrix by comparing the rows of the target matrix with that of the actual matrix and returns four parameters (Table 1) including True Negative (TN), True Positive (TP), False Negative (FN), and False Positive (FP).

(8) $$\begin{array}{r} Sensitivity\;\left(\%\right)\;=\;100\times TP/\left(TP+FN\right) \end{array}$$

(9) $$\begin{array}{r} Specificity\;\left(\%\right)\;=\;100\times TN/\left(TN+FP\right) \end{array}$$

**Table 1:** Causal interactions parameters and explanation.

**Parameter**	**Description of parameter**
TN	TN denotes the number of direct interactions that were not available and were truly marked as non-existent.
TP	TP describes causal interactions that were available and truly labeled as existent.
FN	FN denotes the number of causal interactions that were incorrectly marked as not existing.
FP	FP denotes the number of directed interactions that were incorrectly marked as existing or indicates the number of pairs that were identified to have false causal relationships.

The adjacency matrix linkage bias and network patterns are estimated using the GCA, BVTE, and MTE under various SNR conditions. Based on the simulated networks, we also compute the edges recovered and the adjacency matrix linkage bias. Adjacency matrix linkage bias can be defined as follows:

(10) $$\begin{array}{r} \Delta Y=\frac{∥Y_{c}-Y_{b}∥}{∥Y_{c}∥} \end{array}$$

where *Y*_*c*_ is the adjacency matrix linkage estimated without any added noise effect, and *Y*_*b*_ is the corresponding parameter subjected to noise condition.

We also evaluated the strength of the networks produced by GCA, BVTE, and MTE by considering the total number of edges in the network. The 8 nodes network comprises 56 causal linkages, those edges with directed causal consistent with the originally defined edges are described as correct linkages.

#### Simulation Performance

**Figure 3:**
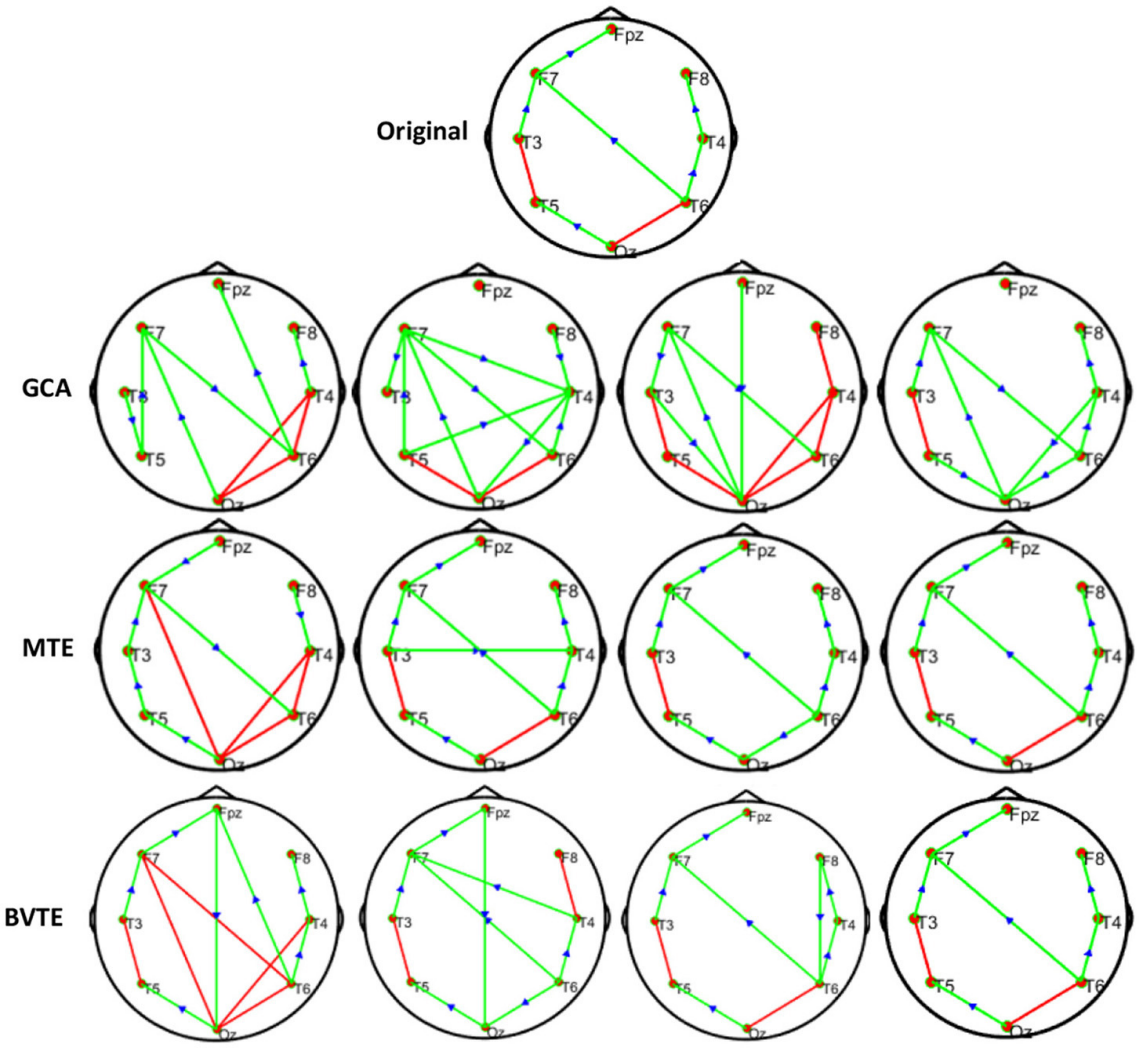
Original or predefined 8 nodes simulated network and estimated non-linear networks by GCA, MTE, and BVTE with (*r* = *f* (*x*), $r=\frac{\left(2.40\times 9x\right)}{1+\text{exp}\left(-4x\right)}$).

**Figure 4:**
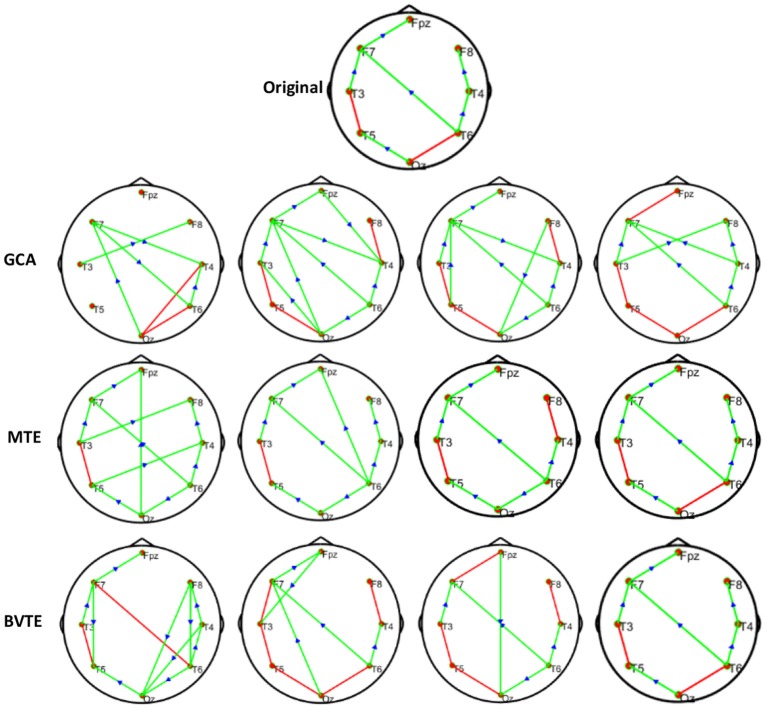
Original or predefined 8 nodes simulated network and estimated non-linear networks by GCA, MTE, and BVTE with *r* = *S* (*x*), $r=\frac{1}{\left(1+\text{exp}\left(-x\right)\right)}$.

**Figure 5:**
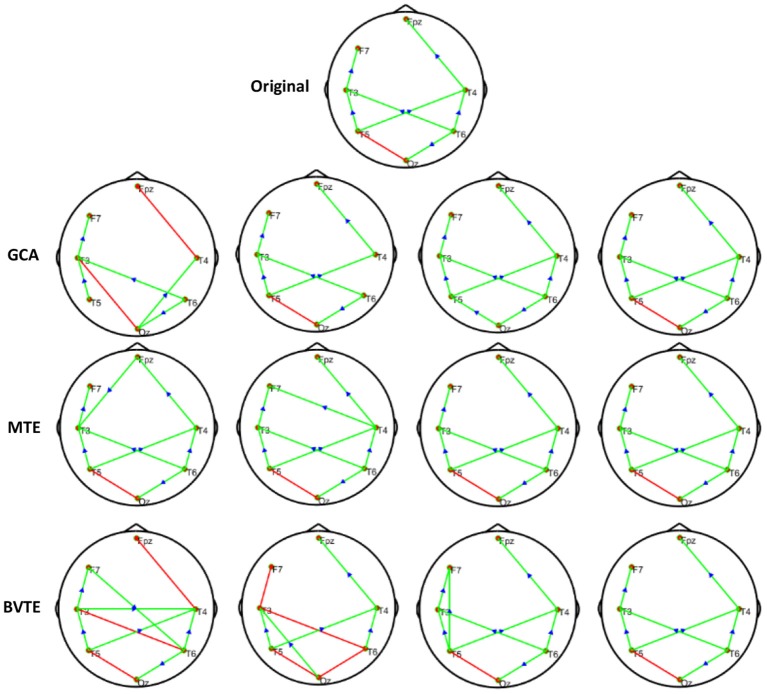
Original or predefined 7 nodes simulated network and estimated linear networks by GCA, MTE, and BVTE with *Y* = *A* × *B*.

**Figure 6:**
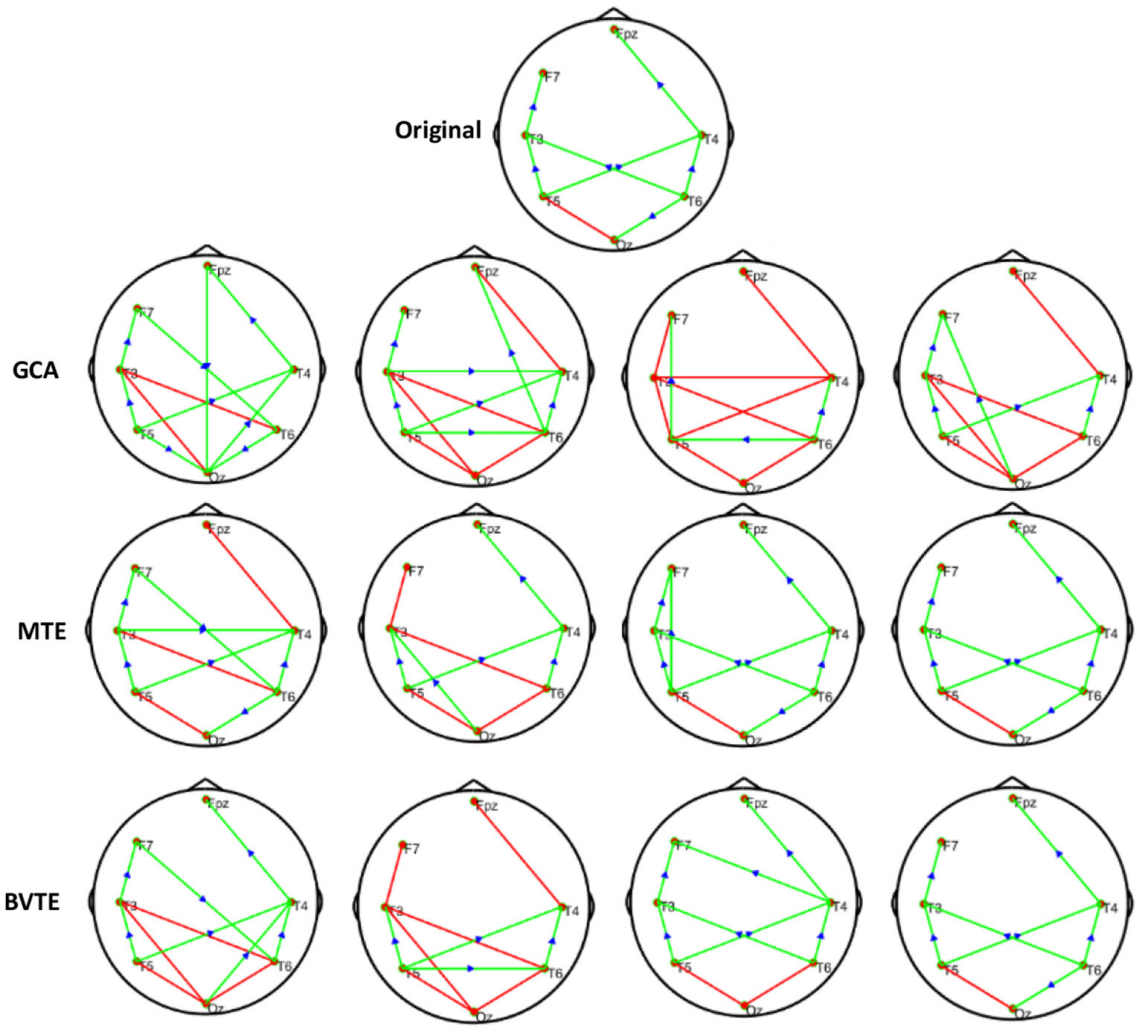
Original or predefined 7 nodes simulated network and estimated non-linear networks by GCA, MTE, and BVTE with (*r* = *f* (*x*), $r=\frac{\left(2.40\times 9x\right)}{1+\text{exp}\left(-4x\right)}$).

**Figure 7:**
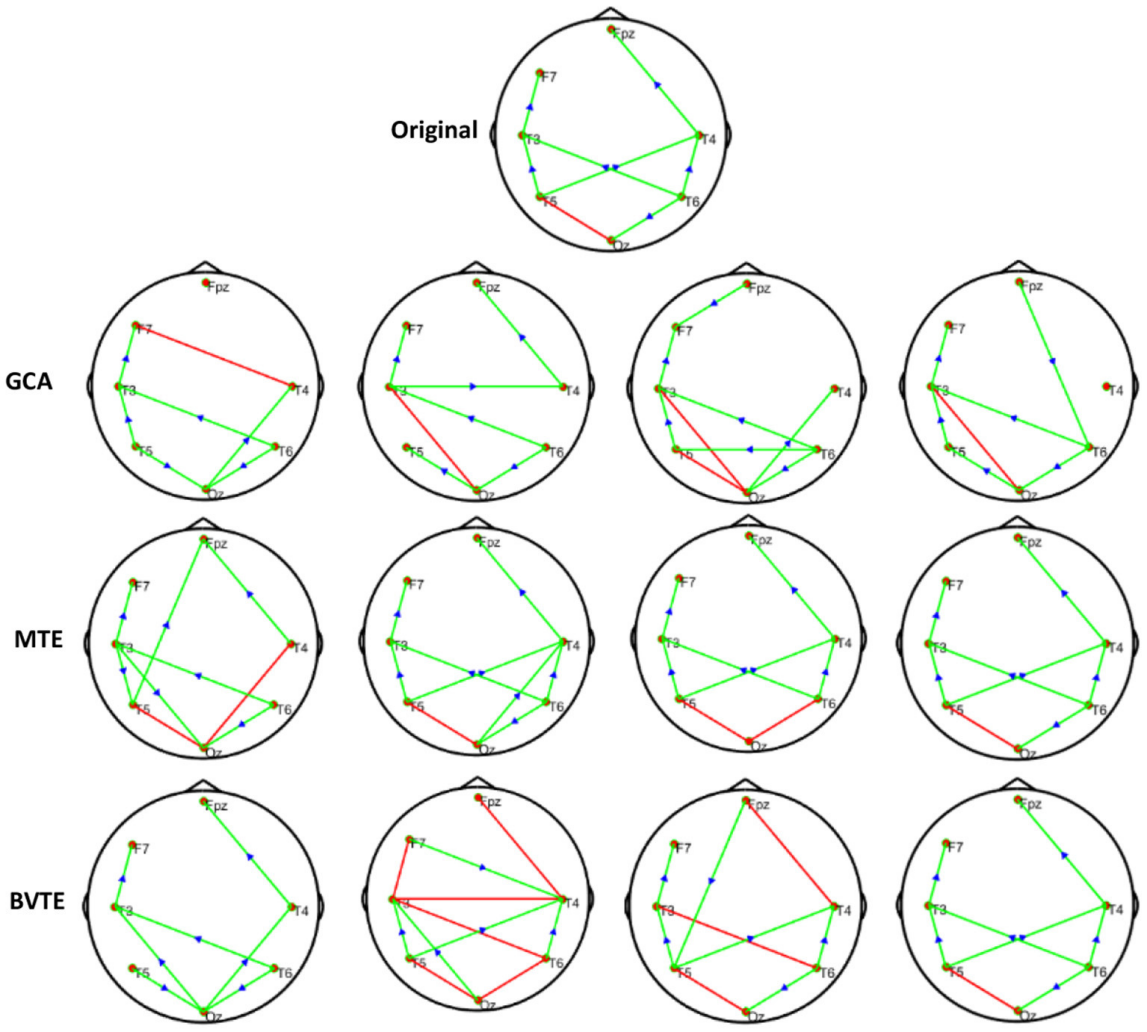
Original or predefined 7 nodes simulated network and estimated non-linear networks by GCA, MTE, and BVTE with *r* = *S* (*x*), $r=\frac{1}{\left(1+\text{exp}\left(-x\right)\right)}$.

As displayed in Figures 2, 5, under the linear condition, under most cases, the GCA, MTE, and BVTE could correctly estimate the network structures (Figures 2, 5), respectively just the same with the original or predefined ones. Unfortunately, under the various non-linear conditions of varied SNRs, the GCA failed to capture the predefined network structure (Figures 3, 4, 6, 7). In contrast, the MTE outperformed the GCA and BTE. Figures 3, 4, 6, 7 depict two of the non-linear simulation conditions(*r* = *f*(*x*),$r=\frac{\left(2.40\times 9x\right)}{1+\text{exp}\left(-4x\right)}$), *r* = *S*(*x*),$r=\frac{1}{\left(1+\text{exp}\left(-x\right)\right)}$, estimated by GCA, MTE and BVTE, respectively. These figures are similar to the other three non-linear simulation conditions. All the simulated figures have the similar structure, which includes original, GCA, MTE, and BVTE results. Besides, the results from left to right are under the SNR of −10, −5, 5, and 10 dB, by row, respectively. In each figure, the green arrows show unidirectional causal interactions and the red lines depict bidirectional connections.

To further demonstrate the advantages of MTE on the network edges recovery over GCA and BVTE, we added few more networks to the already demonstrated figures in Figures 2–4 by simulating additional 7 nodes with networks of structures different from that in Figures 2–4. This is shown in Figures 5–7. It could be noticed from the figures again that MTE was able to recover the network edges better than GCA and BVTE both in linear and non-linear states.

Thereafter, Tables 2, 3 quantitatively display the performances of the average results from 200 runs with parameters of adjacency matrix linkage bias, edges recovered, sensitivity, and specificity under varied SNRs on the 8 nodes simulation. The values highlighted depict the estimator or method which had the least adjacency matrix linkage bias, the highest consistent linkage edges or recovery edges, and also the highest sensitivity and specificity. Out of the six simulations, the MTE outperformed the GCA and BVTE in both linear and non-linear conditions, which is validated by the independent paired *t*-test with a significance level of 0.05.

**Table 2:** A consistent number of edges recovered by GCA, BVTE, and MTE methods.

**Causal relationship function and description**	**Linear/Non-linear**	**Gaussian noise SNR (dB)**	**GCA**		**BVTE**		**MTE**	
			**Bias**	**Edges recovered**	**BVTE bias**	**Edges recovered**	**Bias**	**Edges recovered**
*Y* = *A* × *B*	Linear	−10	0.98 ± 0.09	48.01 ± 1.08	0.99 ± 0.08	47.86 ± 1.09	0.61 ± 0.13	53.37 ± 0.79
		−5	0.95 ± 0.07	48.81 ± 1.04	0.97 ± 0.05	48.74 ± 1.06	0.59 ± 0.12	53.39 ± 0.78
		5	0.75 ± 0.05	51.22 ± 1.01	0.76 ± 0.04	50.65 ± 1.03	0.48 ± 0.11	53.58 ± 0.67
		10	0.62 ± 0.02	53.36 ± 0.78	0.65 ± 0.02	52.78 ± 0.80	0.36 ± 0.09	54.12 ± 0.41
*r* = *C*(*x*) *r* = cos(*x*)+sin(*x*)	Non-linear	−10	0.99 ± 0.08	38.81 ± 3.23	0.98 ± 0.05	42.95 ± 3.15	0.64 ± 0.08	44.58 ± 0.10
		−5	0.97 ± 0.06	38.91 ± 3.03	0.82 ± 0.03	45.97 ± 3.01	0.60 ± 0.07	47.99 ± 0.07
		5	0.78 ± 0.04	39.98 ± 3.01	0.73 ± 0.03	48.99 ± 2.47	0.52 ± 0.04	50.18 ± 0.04
		10	0.72 ± 0.01	42.28 ± 2.88	0.64 ± 0.02	50.13 ± 2.70	0.51 ± 0.02	51.30 ± 0.01
*r* = *f*(*x*) $r=\frac{\left(2.40\times 9x\right)}{1+\text{exp}\left(-4x\right)}$	Non-linear	−10	0.98 ± 0.08	42.58 ± 2.55	0.78 ± 0.08	44.89 ± 2.43	0.58 ± 0.09	47.99 ± 0.12
		−5	0.95 ± 0.07	45.69 ± 2.48	0.65 ± 0.05	47.67 ± 2.22	0.55 ± 0.07	49.68 ± 0.10
		5	0.69 ± 0.03	47.82 ± 2.58	0.58 ± 0.02	49.32 ± 2.14	0.53 ± 0.04	51.04 ± 0.08
		10	0.63 ± 0.02	49.21 ± 2.45	0.54 ± 0.01	51.16 ± 1.25	0.52 ± 0.02	52.06 ± 0.05
*r* = cos*inusoidal*(*x*) *r* = cos(2π*x*)	Non-linear	−10	0.99 ± 0.05	38.78 ± 3.33	0.73 ± 0.08	46.18 ± 3.29	0.67 ± 0.14	48.96 ± 0.25
		−5	0.89 ± 0.04	43.71 ± 2.25	0.67 ± 0.03	47.71 ± 2.55	0.65 ± 0.13	48.99 ± 0.24
		5	0.71 ± 0.02	45.01 ± 2.20	0.59 ± 0.02	48.01 ± 2.10	0.62 ± 0.11	49.70 ± 2.03
		10	0.68 ± 0.01	46.86 ± 2.17	0.56 ± 0.01	50.86 ± 1.13	0.59 ± 0.07	51.42 ± 0.01
*r* = *H*(*x*) *r* = exp(sin(2π*x*))	Non-linear	−10	0.99 ± 0.08	44.79 ± 1.79	0.68 ± 0.06	46.99 ± 0.32	0.69 ± 0.15	48.97 ± 0.29
		−5	0.98 ± 0.03	44.99 ± 0.99	0.67 ± 0.04	47.87 ± 0.11	0.68 ± 0.14	48.99 ± 0.09
		5	0.70 ± 0.02	46.28 ± 0.89	0.64 ± 0.02	48.23 ± 0.72	0.61 ± 0.10	50.02 ± 0.05
		10	0.59 ± 0.01	46.99 ± 0.61	0.59 ± 0.10	49.89 ± 0.45	0.58 ± 0.07	51.88 ± 0.02
*r* = *S*(*x*) $r=\frac{1}{\left(1+\text{exp}\left(-x\right)\right)}$	Non-linear	−10	0.97 ± 0.09	47.55 ± 1.14	0.63 ± 0.07	47.67 ± 0.38	0.66 ± 0.11	48.98 ± 0.74
		−5	0.95 ± 0.07	48.32 ± 1.12	0.61 ± 0.03	49.42 ± 0.15	0.60 ± 0.09	50.12 ± 0.68
		5	0.79 ± 0.05	49.34 ± 1.08	0.58 ± 0.09	49.78 ± 0.60	0.54 ± 0.07	51.03 ± 0.20
		10	0.56 ± 0.02	49.99 ± 0.06	0.55 ± 0.07	51.88 ± 0.23	0.52 ± 0.04	52.45 ± 0.18

**Table 3:** Sensitivity and specificity analysis by GCA, BVTE, and MTE methods.

**Causal relationship function and description**	**Linear/Non-linear**	**Gaussian noise SNR(dB)**	**GCA**		**BVTE**		**MTE**	
			**Sensitivity**	**Specificity**	**Sensitivity**	**Specificity**	**Sensitivity**	**Specificity**
*Y* = *A* × *B*	Linear	−10	89.59 ± 9.61	87.33 ± 2.62	85.53 ± 9.74	86.92 ± 2.62	91.92 ± 7.12	88.57 ± 5.69
		−5	92.64 ± 4.56	89.45 ± 5.66	89.74 ± 7.66	87.67 ± 5.71	93.98 ± 4.34	90.75 ± 3.32
		5	94.72 ± 7.70	92.57 ± 6.72	93.83 ± 6.50	91.71 ± 7.43	95.88 ± 4.20	93.79 ± 5.51
		10	95.98 ± 3.56	94.81 ± 5.52	94.87 ± 4.64	93.63 ± 7.33	97.99 ± 2.33	96.49 ± 3.61
*r* = *C*(*x*) *r* = cos(*x*)+sin(*x*)	Non-linear	−10	68.34 ± 14.59	74.58 ± 5.34	85.34 ± 4.31	84.87 ± 5.56	88.54 ± 1.14	88.21 ± 2.42
		−5	75.52 ± 7.31	76.88 ± 6.43	87.43 ± 6.23	88.12 ± 4.40	91.49 ± 1.07	91.98 ± 2.32
		5	83.74 ± 5.17	84.89 ± 3.26	90.61 ± 7.30	91.77 ± 4.17	93.91 ± 1.50	94.67 ± 3.12
		10	91.07 ± 2.48	92.33 ± 1.16	93.11 ± 3.50	94.04 ± 1.82	96.01 ± 0.15	96.99 ± 1.04
*r* = *f*(*x*) $r=\frac{\left(2.40\times 9x\right)}{1+\text{exp}\left(-4x\right)}$	Non-linear	−10	48.84 ± 2.41	72.68 ± 5.72	76.94 ± 3.54	82.87 ± 4.78	79.51 ± 3.86	85.96 ± 3.68
		−5	51.92 ± 1.39	78.96 ± 3.47	83.96 ± 2.87	87.31 ± 4.60	86.78 ± 2.91	89.10 ± 5.71
		5	69.98 ± 2.89	87.88 ± 4.87	89.93 ± 1.78	87.72 ± 5.13	93.78 ± 0.98	91.09 ± 3.77
		10	74.69 ± 2.76	91.21 ± 1.87	93.91 ± 2.19	90.88 ± 1.40	95.04 ± 1.58	93.16 ± 0.83
*r* = cos*inusoidal*(*x*) *r* = cos(2π*x*)	Non-linear	−10	40.22 ± 21.25	82.40 ± 4.48	47.69 ± 2.71	83.54 ± 3.63	52.83 ± 1.28	87.67 ± 3.56
		−5	48.78 ± 2.96	89.31 ± 5.69	64.35 ± 1.50	90.09 ± 0.14	66.14 ± 0.89	92.18 ± 1.16
		5	67.09 ± 4.58	93.17 ± 0.97	85.95 ± 5.66	91.08 ± 0.30	88.29 ± 4.09	93.99 ± 2.21
		10	74.42 ± 2.84	94.98 ± 1.78	90.87 ± 1.11	92.20 ± 0.61	93.14 ± 0.89	94.58 ± 5.10
*r* = *H*(*x*) *r* = exp(sin(2π*x*))	Non-linear	−10	49.71 ± 17.20	69.36 ± 4.66	69.88 ± 2.54	86.12 ± 0.41	72.26 ± 1.42	87.85 ± 1.28
		−5	57.12 ± 6.53	71.06 ± 1.77	84.63 ± 2.20	86.92 ± 0.13	86.07 ± 1.50	88.96 ± 0.88
		5	68.09 ± 3.36	74.91 ± 0.82	86.12 ± 4.14	88.98 ± 0.94	89.81 ± 0.23	91.74 ± 1.65
		10	77.82 ± 5.63	79.99 ± 0.74	91.90 ± 1.72	93.87 ± 2.19	94.51 ± 1.11	95.83 ± 1.32
*r* = *S*(*x*) $r=\frac{1}{\left(1+\text{exp}\left(-x\right)\right)}$	Non-linear	−10	34.85 ± 7.86	66.87 ± 2.85	70.91 ± 3.13	83.73 ± 1.77	73.18 ± 2.63	86.98 ± 2.84
		−5	42.74 ± 5.83	71.93 ± 4.59	76.89 ± 1.25	86.42 ± 3.87	79.99 ± 0.82	88.79 ± 4.63
		5	53.42 ± 6.73	76.89 ± 2.96	85.33 ± 0.84	87.75 ± 4.44	87.78 ± 1.14	90.03 ± 3.50
		10	74.38 ± 7.42	79.61 ± 1.13	88.15 ± 2.37	89.56 ± 1.41	91.27 ± 1.08	92.11 ± 0.24

### Real P300 EEG

#### Participants

This experiment included 48 right-handed (self-reported) participants, which consisted of 23 SCZ patients (10 females, age 28.87 ± 7.68) and 25 HCs (11 females, age 29.44 ± 5.75). All participants had the normal or corrected-to-normal vision. None of them had used any medication, and there had been no personal or family history of psychiatric or neurological disease. The Ethics Committee of Peking University Sixth Hospital approved this study. Before experiments, all participants gave the written informed consent with their names signed on it.

#### Experimental Protocol

**Figure 8:**
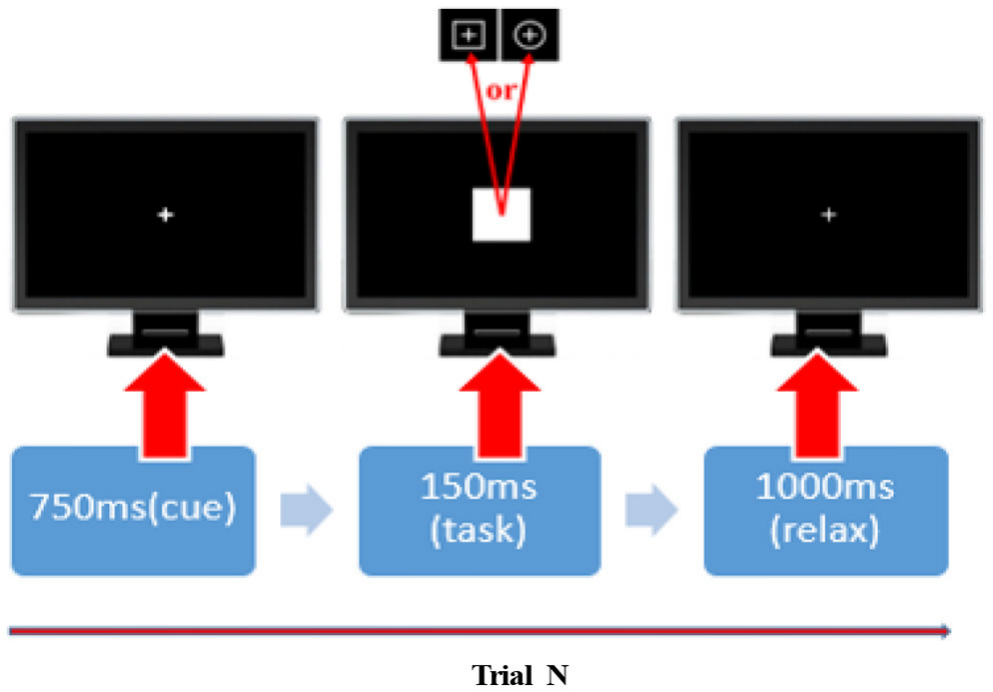
The timeline of a given P300 trial. In each P300 trail, a 750-ms cue, 150-ms stimulus, and 1,000-ms break were added. The squares and circles with a thin cross in the center represent the standard and target stimuli in that order.

Before the commencement of the experiment, all participants were instructed to be seated comfortably, stay relaxed and were also asked to control their eye blinks and body movements in the experiments. A square with a thin cross in the center and a circle with a thin cross in the center were defined as the standard and target stimulus, respectively. We included a 5-min, eye-closed resting-state session and four runs of P300 tasks during the experiments. In each P300 run, a total of 100 stimuli, 80 standards, and 20 targets, were randomly presented on the computer screen. Figure 8 depicts the timeline of a given P300 trial. In detail, a bold-cross cue was first presented and lasted 750 ms to warn participants to focus their attention and to inform them that a standard (or target) stimulus would appear very soon. Either a standard or target stimulus then appeared on the screen for 150 ms. Participants were asked to press the “1” key on a standard keyboard when they noticed a target stimulus appeared at the same time. A 1,000-ms break was given after and the next trial began.

#### EEG Recording

We recorded the EEG datasets with the Symtop amplifier (Symtop Instrument, Beijing, China) and a 16-channel Ag/AgCl (i.e., Fp1, Fp2, F3, F4, C3, C4, P3, P4, O1, O2, F7, F8, T3, T4, T5, and T6) electrode cap (BrainMaster, Inc., Shenzhen, China). We positioned all the electrodes used in accordance with the 10–20 international electrode placement system and digitized with a sampling rate of 1,000 Hz and online bandpass filtered at 0.05–100 Hz. Electrode AFz was used as the reference and was grounded during online recording. The total impedance during the whole task of all electrodes was kept below 5 *K*Ω, during the recording.

#### Effective Network

Since, we aimed to investigate the brain network deterioration of the SCZ in the oddball task, in this study, only the EEG datasets of the four runs of P300 tasks were included in the following analyses. To construct an effective network, we used multiple standard procedures to preprocess the task datasets. The multiple procedures comprise [0.5 Hz, 30 Hz] offline bandpass filtering, 1-s length data segment (ranging from 200 ms before and 800 ms after targets onset [−200 ms, 800 ms]), [−200 ms, 0 ms] baseline correction, artifact-trial removal using a threshold of ±100 μv, and Reference Electrode Standardization Technique (REST). Thereafter, based on the EEG time series we generated, the GCA, MTE, and BVTE were used to construct the corresponding weighted effective network for the HC and SCZ.

The effective network is a square asymmetric adjacency matrix where the number of rows and columns is equal to the number of electrodes. The GCA, MTE, and BVTE are then applied to estimate the adjacency matrix per task trial per subject. Thereafter, the final weighted rest (also task), a 16 × 16 adjacency matrix, directed brain network for each subject was acquired by averaging matrices across all artifact-free segments (also task trials), and eventually, we conducted independent *t*-test to unearth the potential difference (*p* < 0.05) in the brain networks of HC and SCZ for both methods.

#### Topological Differences in HC and SCZ

**Figure 9:**
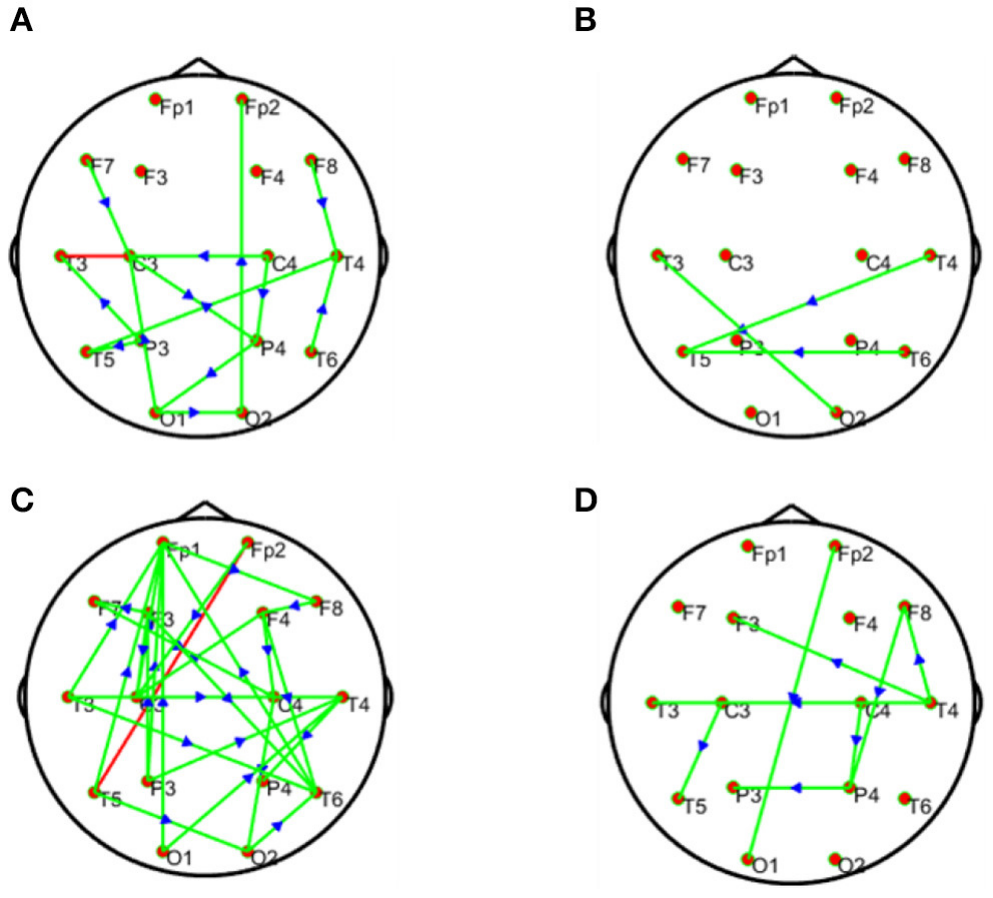
Statistical analysis for the differential network topology between the HC and SCZ estimated by the GCA **(A,B)** and MTE **(C,D)**. The first column depicts that the connectivity of HC is stronger than that of SCZ, whereas the second column depicts that the connectivity of SCZ is lesser or weaker than that of HC. In each subfigure, the red and green lines depict bidirectional and unidirectional connectivity, respectively.

Figure 9, visually demonstrates differential network topology between HC and SCZ (*P* < 0.05, FDR corrected) estimated by the methods-GCA and MTE. As displayed in Figure 9, the GCA (Figures 9A,B) and MTE (Figures 9C,D) showed much denser connectivity for the HC, compared to that of the SCZ, which extended on the frontal and parietal lobes. In specific, the corresponding stronger and denser causal connectivity can be found to flow from prefrontal/frontal to parietal lobes. In addition, compared to the GCA, the MTE gives more causal linkages, shows the dense edges in the frontal lobe.

#### Statistical Comparison for the Topographical Difference Between HCs and SCZ Patients

**Figure 10:**
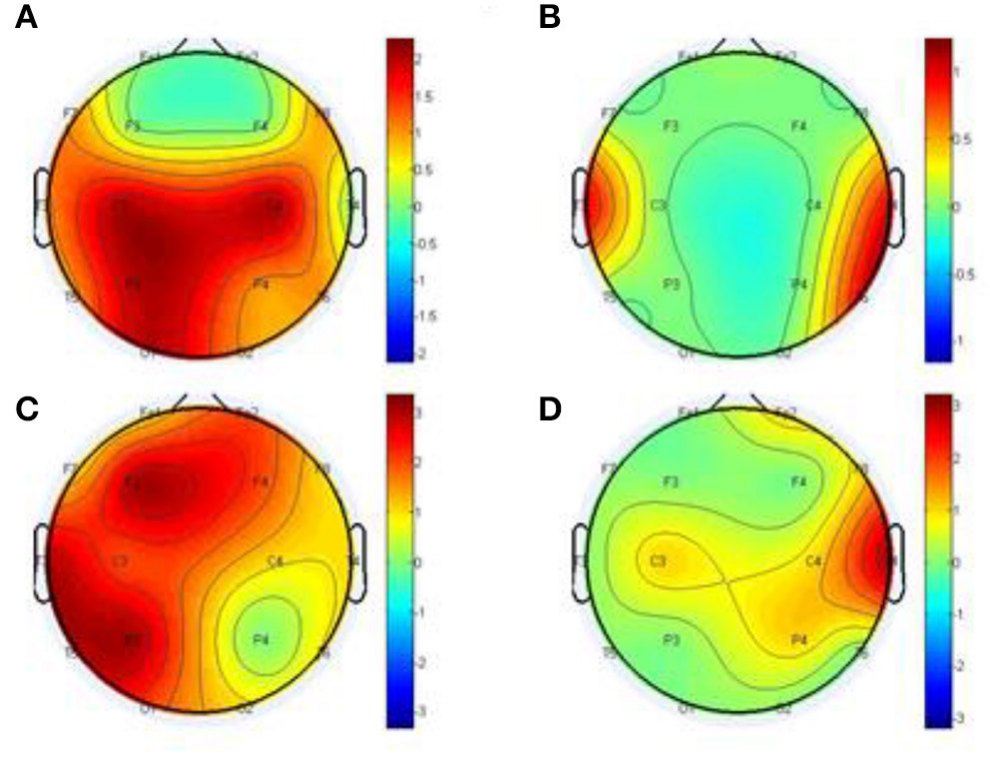
Statistical comparison for the topographical difference between HCs and SCZ using out degree, estimated by the GCA **(A,B)** and MTE **(C,D)**. The first column depicts the connectivity of HC is stronger than that of SCZ, whereas the second column depicts the connectivity of SCZ is weaker than that of HC.

We conducted further analysis on Figure 9 to prove our method MTE over the GCA using out degree in Figure 10. The node out-degree can be defined as the number of edges pointing out or going out of the node. The number of edges connecting the node with any or all other nodes is termed Node degree. If the nodes are more connected, it means they have greater degree and vice versa (Fornito et al., 2016). The degree of a node could be in-degree or out-degree. For example^1^ in directed network, if we have an edge with a path from node i to node j, then Node i's out-degree is ∑_*jgij*_.

This has important influence on the brain network. This information flow can influence the properties of dynamical systems that evolve on the brain network, such as the synchronization of networked oscillators. Moreover, different nodes play or serve distinct topological roles in the brain network, with highly connected nodes exerting a particularly important influence over network function (Fornito et al., 2016). Thus, in our study after the construction of the differential network topology, we based our analysis on the information flow out of the node to further explain Figure 9.

After the out degree analysis, our proposed method-MTE still proved to be better than the conventional method GCA. In Figure 10, our method proved better because it could help locate the network channels well which the GCA method couldn't. There are significant differences between the HCs and SCZ for all the methods. However, MTE showed more outgoing degrees compared to GCA. The out degree for MTE could help locate the brain regions or channels better than the GCA and with this we could see the nodes which are highly connected and those with less or no connections. In Figure 10, MTE has more variation of information between all the channels compared to GCA. The colors correspond to a variation of information between the regions or channels (Van Den Heuvel and Fornito, 2014; Yang et al., 2017). GCA has the following results for its out degrees for HCs and SCZ patients, respectively:

Channels (Fp1 of HC and Fp1 of SCZ, Fp2 and Fp2, F3 and F3, F3 and F3, F4 and F4), have no difference in their channels. Meanwhile, the channels (F7 and F7, F8 and F8, C4 and C4, T3 and T3, T4 and T4, T5 and T5, T6 and T6, P3 and P3, P4 and P4, O1 and O1, O2 and O2) had a difference between them. The highest out degree for HCs is 2 for the channels- C2, C4, P3, and O1. SCZ patients had 1 as the highest out degree.

For MTE, only the channels (F8 and F8, C4 and C4) had no difference in between them. The channels (Fp1 and Fp1, Fp2 and Fp2, F7 and F7, F3 and F3, F4 and F4, T3 and T3, T4 and T4, C3 and C3, P3 and P3, P4 and P4, T5 and T5, T6 and T6, O1 and O1, O2 and O2) had a difference between their channels. In all, channels F3, P3 T3 and T5 had the highest out degrees for HCs whiles channel T4 also had the highest out degree for SCZ patients (Rubinov and Bullmore, 2013; van Straaten and Stam, 2013). The analysis above clearly show that our method MTE still had the best performance in the out degree condition. It had more information flow from out of the nodes and also more channel influence than the GCA method.

## Discussion

Non-linearity characterizes our daily activities. Biological systems, such as EEG, is linear and inherently non-linear. Although linear methods are important and have obtained satisfying findings in EEG analysis, they compromise the underlying non-linearity characteristics or non-linear causal dynamics. The applications of non-linear methods in EEG analysis will, therefore, pave a way for logical steps that can be used to enhance the characterization of these signals. The GCA has the problem of model dependency, statistical and conceptual problems, and it ignores the system dynamics (Stokes et al., 2018). BVTE analysis also lead to spurious and redundant interactions and may miss synergistic interactions between multiple relevant sources and the target (Wollstadt et al., 2019). In the current study, we thus proposed to apply the MTE to the task EEGs of the SCZ and HC, to investigate the mechanism explaining the cognitive deficits in the SZ, from the perspective of effective connectivity.

GCA computation or estimation encounter many problems. It can either be severely biased or have high variance and these shortcomings lead to spurious, redundant, etc. results. GCA estimation or computation alone are not interpretable without examining the component behaviors of the system model even if these estimations are done correctly and also ignoring the critical components system's dynamics. On the basis of these analysis, the idea or notion of causality quantified is not compatible with the objectives of many neuroscience research investigations and this has led to highly counterintuitive and potentially misleading results with GCA (Stokes et al., 2018). GCA in time domain cannot correctly determine how strongly one time series influences the other especially when there is directional causality between two time series. In other words a larger GCA value does not necessarily mean higher real causality, or vice versa (Hu et al., 2016). Moreover, many connectivity measures like GCA that are based on the autoregressive model do not always reflect true neuronal connectivity (Schindler, 2011). TE was also formulated for the bivariate case; that is between a single source and a single target. However, in a multivariate setting, bivariate analysis may lead to false positive or false negative results inferring spurious or redundant causality or interactions and also missing synergistic interactions between important sources and the target. Usually, these many sources together send more information into the target than what could be detected from examining source contributions individually (Tanaka et al., 2013; James et al., 2016; Wollstadt et al., 2019). These findings are confirmed by our study in Tables 2, 3 and Figures 2–7, 9, 10, especially the networks revealed by the methods on the real data.

The MTE could detect both linear and non-linear signals better than the GCA and the BVTE and is able to account for all relevant sources of a target. By predefining the simulated network structure as well as the corresponding time courses, we applied the GCA, BVTE, and MTE methods to estimate the defined flow matrix and the directed networks under the influence of Gaussian noise in order of −10, −5, 5, and 10 dB, and evaluated the performance of the GCA, BVTE, and MTE under adjacency matrix linkage bias, edges recovered, sensitivity, and specificity. Figures 2, 5 demonstrate that the GCA, MTE, and BVTE have the potential for effectively estimating the originally defined network patterns under the linear condition of varied SNRs, respectively. However, as displayed in Figures 3, 4, 6, 7 corresponding to two of the various non-linear conditions, the GCA was not able to recover the original defined network patterns and produced many false linkages. Even though, BVTE was able to recover the predefined network but in contrast, the MTE outperforms the GCA and the BVTE under same conditions (Figures 3, 4, 6, 7). The MTE is able to suppress the turbulent noise contaminated and efficiently estimated most of the original or predefined network linkages, which is unlike the GCA affected by the noise and thus performed badly. Specifically, the strength of edges recovered and the reduction of edges strength with bias errors, sensitivity, and specificity are shown in Tables 2, 3 which reveals clearly how these three methods are influenced by noise in linear and non-linear conditions. With consistency, MTE always held a good performance in all the functional indexes with less or lowest bias errors to GCA and BVTE in a mean of 200 runs. That is, in the linear and five non-linear simulations under all the SNR conditions, the MTE could recover highest linkages closed to the predefined network structure, compared to the GCA and BVTE, as well as the highest sensitivity and specificity. As illustrated previously, the MTE is capable of overcoming spurious or redundant interactions and is also able to reveal synergistic interactions between multiple relevant sources that the GCA and BVTE lack. The topological differences between the three methods indeed show clearly that the MTE method could estimate the networks better than the GCA and BVTE both in the simulation and the real task EEG computation.

A research by Bassett and Bullmore (2009) reported that the causal interactions between the components of the prefrontal-limbic system determines the global trajectories of the individual's brain activation, with the strengths and modulations of these causal interactions being potentially key components determining or underlying the differences between HC individuals and those with SCZ. Research also has it that SCZ patients have significant reduction in strength of functional connectivity and increased diversity of functional linkages. Meanwhile topologically, functional brain network has a reduction on clustering and small-worldness, probability of high-degree hubs, but increased robustness in the SCZ group. The medial parietal, premotor and cingulate, and right orbitofrontal cortical nodes of functional networks in SCZ also locally saw a reduction in degree and clustering (Lynall et al., 2010). A research conducted in Jalili and Knyazeva (2011) and Ray et al. (2017) indicated that many higher deficits in cognition in SCZ may be as a result of dysfunction of cognitive control deficits in SCZ. In a comparative analysis between SCZ and HCs, SCZ individuals demonstrated a reduced activation in the dorsolateral prefrontal cortex (DLPFC), ventrolateral prefrontal cortex (VLPFC), dorsal anterior cingulate cortex (ACC), pre-SMA, ventral premotor cortex, posterior areas in the temporal and parietal cortex, and sub-cortical areas. Further meta-analysis also revealed disrupted and decreased resting-state functional connectivity (rsFC) within the self-referential network and default mode network which play roles in the malfunction of information processing in SCZ, while the core network might act as a dysfunctional hub of regulation (Li S. et al., 2019). These meta-analysis results are consistent with our present studies in Figures 9, 10.

Based on our analysis and other findings, SCZ patients most often find it difficult to retain their attention during tasks unlike the HC. Usually the altered brain regions affect the information processing in the SCZ and these disruptions give rise to P300 malfunctions, which eventually disturbs the brain at rest in terms of abnormalities (Li F. et al., 2019). As a result of the malfunctioning of neurotransmitters, the ability of the SCZ patients to perceive reality is dumped (Karlsgodt et al., 2010; Alonso-Solís et al., 2015). In fact, people living with psychiatry or mental problems have severe brain network deterioration (Fogelson et al., 2014). The disruption of large-scale brain regions can largely account for the dysfunction of brain function in people living with the SCZ, and this disruption of the interregional connection may give rise to failure of the functional integration in the SCZ, thus paving a way for proper explanation of the abnormal behavior and cognitive impairment in patients with the SCZ (McKiernan et al., 2014; Zhang et al., 2019). Our findings in Figures 9, 10 indeed show the differential network topology and its comparison which show clearly the complete disruption of the multiple brain regions of the SCZ in relation to the HC agreeing with these studies. In specific, the HC showed the denser connectivity compared to that of the SCZ and these connections are extended on the frontal and parietal lobes. In essence, an alteration in causal connectivity between parts of the prefrontal cortex and the limbic system is found in Menon (2011), Qiu et al. (2014). The prefrontal cortex, the basal ganglia, and limbic system, etc. are interconnected and hence an attack of infection on one region will eventually affect the others. These above considerations drive us to conclude that the directed causal connectivity from prefrontal/frontal to parietal lobes is deteriorated, which then leads to the deficits in the P300, e.g., decreased P300 amplitudes.

Specifically, Figures 2–7, 9, 10 again show clearly that the MTE method could estimate the networks better than the GCA and BVTE not only in the simulation (Figures 2–7, Tables 2, 3), but also in the real EEG application with GCA in Figures 9, 10. It holds its superiority over the GCA and BVTE in simulation and with GCA in real EEG analyses by giving a more satisfying performance. Our study and other studies (Gourévitch et al., 2006; Liu and Aviyente, 2012) have found that the GCA is not robust enough in detecting non-linear linkages but it seems to be effective in detecting linear linkages. Also though BVTE could detect the non-linear causality better than GCA, in contrast, the MTE can address this problem. The MTE is able to handle spurious or redundant interactions and also unearth synergistic interactions between multiple relevant sources (Stokes et al., 2018; Wollstadt et al., 2019). Thus, when exploring the brain network deterioration in the SCZ patients, the MTE indeed outperforms the GCA and BVTE and seems to be a good choice.

## Conclusion

In summary, we testified to the fact that non-linear dynamics can give clearer information for better understanding of the causal dynamic issues surrounding EEG signals when it comes to its inherent non-linearity. Compared to the GCA and BVTE, the MTE was remarkably helpful in marking the causality either in a linear or non-linear system, which uncovered the brain dysfunction in effective connectivity for the SCZ that is deteriorated at the frontal and parietal lobes.

## Data Availability Statement

The datasets generated for this study are available on request to the corresponding author.

## Ethics Statement

The studies involving human participants were reviewed and approved by Peking University Sixth Hospital. The patients/participants provided their written informed consent to participate in this study.

## Author Contributions

PX, JW, and WD conceived of and designed the experiments. JW performed the experiments. DH, CL, and YL analyzed the dataset. DH, FL, and PX wrote the manuscript. CL, WA, JB, and DY provided some useful suggestions in manuscript writing.

### Conflict of Interest

The authors declare that the research was conducted in the absence of any commercial or financial relationships that could be construed as a potential conflict of interest.

## Footnotes

^1^Network/Graph Theory Graph-based representations Protein-Protein Interaction.
